# CD38-NAD^+^-Sirt1 axis in T cell immunotherapy

**DOI:** 10.18632/aging.102385

**Published:** 2019-10-23

**Authors:** Shilpak Chatterjee, Paramita Chakraborty, Shikhar Mehrotra

**Affiliations:** 1 Indian Institute of Chemical Biology, TRUE Campus, Kolkata 700091, India; 2 Department of Surgery, Hollings Cancer Center, Medical University of South Carolina, Charleston, SC 29425, USA

**Keywords:** T cell, immunotherapy, melanoma, metabolism

Harnessing the cytotoxic ability of T cells is emerging as a powerful therapeutic intervention against various malignancies. In past few years remarkable clinical successes have been achieved in treating cancer patient’s by bolstering the anti-tumor T cell response either by adoptively transferring tumor epitope specific TCR or CAR engineered T cells or by antibody mediated blockade of co-inhibitory immune checkpoints (PD1, CTLA4, Lag3, Tim3) on tumor reactive T cells [1]. While these therapies benefit a fraction of patients with malignancy, majority of them either do not respond to these therapies or experience tumor relapse after initial response due to T cell dysfunction in the tumor microenvironment. Therefore, understanding the T cell intrinsic factors that would instill tumor reactive T cells with a stable functional state at the tumor site is warranted.

Recent studies have demonstrated that the T cell dysfunctionality in tumors is a dynamic process, which involves remodeling of the epigenetic pathways that lead to a compact chromatin state and reduced transcription of genes associated with effector function [2]. Additionally, the cellular energetic pathway is also a key feature of the unresponsive state of anti-tumor T cells. Most intriguingly, mitochondrial quality and integrity that ensures efficient oxidative phosphorylation (OXPHOS) appears to be decisive in regulating the durability and ability of the anti-tumor T cells to respond to immunotherapeutic regimen [3]. However, what connects these seemingly unrelated cellular events of attaining a distinct chromatin and metabolic state that reinforces the stable dysfunction in anti-tumor T cells.

**Figure 1:**
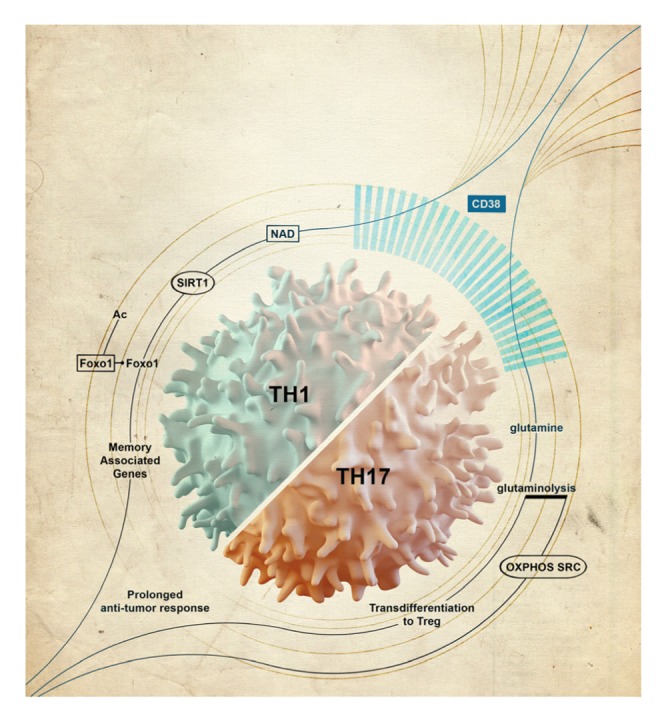
The summary of observation illustrating the unique immunometabolic phenotype of *ex vivo* generated hybrid TH1/TH17 cells that exhibit reduced CD38 expression, high NAD and long-term tumor control upon adoptive transfer.

Recently, using tumor epitope reactive T cells that were *ex vivo* programmed to secrete both IFN-γ and IL17 at the time of adoptive transfer, we identified that CD38-NAD^+^-Sirt1 axis acts as a key determinant of the therapeutic efficacy of anti-tumor T cells [4] (Figure 1). CD38 is an ectonucleotidase with NADase activity highly expressed on tumor infiltrating lymphocytes (TILs), and its expression increases as tumor progresses. T cells with genetic ablation of CD38 not only exhibited complete remission of tumor growth but also improved the durability of the response. Most, notable feature observed in T cells with CD38 deficiency was its preferential reliance on glutaminolysis to empower OXPHOS. We believe that this metabolic reprogramming is critical to sustain the effector function of the anti-tumor T cells in the glucose deprived tumor microenvironment. It is also plausible that dependence on glutaminolysis not only provides the bioenergetic needs but also enables the T cells to harbor a discrete epigenetic landscape owing to the increased production of alpha-ketoglutarate, an epigenetic modifier, and hence instill the T cells with the ability to maintain the stable effector function in the tumor milieu.

In addition to facilitate glutaminolysis, CD38 deficiency in T cells resulted in elevated intracellular level of nicotinamide adenosine dinucleotide (NAD^+^), a metabolite that acts as a substrate for *Sirtuin* (Sirt) family of enzymes catalyzing deacetylation. Numerous studies have implicated the role of Sirt1 in epigenetic modification through targeting different histone marks including H3K9Ac, H4K16Ac and H1K26Ac. In fact, increased histone acetylation at H3K9 (H3K9Ac), which is considered as active transcriptional mark, was reported in *EOMES*, *PRF1*, and *GZMB* loci of memory CD8 T cells. In addition, Sirt1 can also regulates the activity of various transcription factors including TP53, NF-kB, FOXO3a and FOXO1. We observed that in anti-tumor T cells, the transcriptional activity of FOXO1, which has shown to regulate the expression of various T cell memory associated genes including *TCF7*, *Bcl6* and *β-catenin*, was dependent on Sirt1 mediated deacetylation. Therefore, Sirt1 mediated epigenetic modification is an active mechanism that regulates immunotherapeutic efficacy of anti-tumor T cells in suppressive tumor microenvironment. Recently, it is reported that the abundance of TCF1^+^PD1^+^ CD8 T cells at the tumor site is predictive of objective response to immunotherapy as these cells retain the characteristics of stem like T cells with prolonged persistence ability [5]. This data strengthens the role of NAD^+^-Sirt1 axis, which inversely corelates with CD38 expression, in epigenetically regulating various transcription factors/molecules that endow T cells with stemness and superior anti-tumor response. Therefore, strategies to target CD38 and its upstream regulators will not only enhance the metabolic fitness of TILs but also result in epigenetic modulation required for durable anti-tumor response. It must be noted that CD38 is also an established cell surface activation marker on T cells that requires the TCR-CD3 complex for signalling [6]. Thus, it is likely that CD38 bridges chronic TCR stimulation with the dynamic changes in epigenetic landscape of a T cell.

The superior anti-tumor response of the CD38 deficient T cells also could be due to their increased antioxidant potential as discern by the elevated expression of various antioxidant genes including *Trx1, Trx2, SOD1, SOD2* and *Nrf2.* Recently, it has been reported that T cells obtained from the tumor site exhibit increased activation of endoplasmic reticulum (ER) stress responsive protein IRE-1α-XBP1 which inversely corelates with the mitochondrial respiration and anti-tumor property of the T cells [7]. Further study in tumor infiltrating dendritic cells (tDC) revealed that intracellular reactive oxygen species (ROS) was critical in promoting the activation of XBP-1 through the generation of lipid oxidation byproducts, 4-hydroxy-trans-2-nonenal (4-HNE). We believe that through maintaining high anti-oxidant capacity, CD38 deficient T cells are not only resistant to ER stress mediated metabolic and functional impairment at the tumor site, but inhibiting CD38 will also reinvigorate replicative senescent anti-tumor T cells, as has been shown in aging models where it leads to enhanced NAD^+^ [8].
